# Obesity in male volcano mice *Neotomodon alstoni* affects the daily rhythm of metabolism and thermoregulation

**DOI:** 10.3389/fnut.2022.963804

**Published:** 2022-08-04

**Authors:** Andrea Herrera-García, Moisés Pérez-Mendoza, Elvira del Carmen Arellanes-Licea, Deisy Gasca-Martínez, Agustín Carmona-Castro, Mauricio Díaz-Muñoz, Manuel Miranda-Anaya

**Affiliations:** ^1^Unidad Multidisciplinaria de Docencia e Investigación, Facultad de Ciencias, Universidad Nacional Autónoma de México, Juriquilla, Querétaro, Mexico; ^2^Facultad de Ciencias Naturales, Universidad Autónoma de Querétaro, Juriquilla, Querétaro, Mexico; ^3^Instituto de Neurobiología, Universidad Nacional Autónoma de México, Juriquilla, Querétaro, Mexico

**Keywords:** *Neotomodon alstoni*, obesity, metabolism, UCP1, thyroxine hydroxylase, body temperature, daily rhythm

## Abstract

The mouse *N. alstoni* spontaneously develops the condition of obesity in captivity when fed regular chow. We aim to study the differences in metabolic performance and thermoregulation between adult lean and obese male mice. The experimental approach included indirect calorimetry using metabolic cages for VO_2_ intake and VCO_2_ production. In contrast, the body temperature was measured and analyzed using intraperitoneal data loggers. It was correlated with the relative presence of UCP1 protein and its gene expression from interscapular adipose tissue (iBAT). We also explored in this tissue the relative presence of Tyrosine Hydroxylase (TH) protein, the rate-limiting enzyme for catecholamine biosynthesis present in iBAT. Results indicate that obese mice show a daily rhythm persists in estimated parameters but with differences in amplitude and profile. Obese mice presented lower body temperature, and a low caloric expenditure, together with lower VO_2_ intake and VCO_2_ than lean mice. Also, obese mice present a reduced thermoregulatory response after a cold pulse. Results are correlated with a low relative presence of TH and UCP1 protein. However, qPCR analysis of Ucp1 presents an increase in gene expression in iBAT. Histology showed a reduced amount of brown adipocytes in BAT. The aforementioned indicates that the daily rhythm in aerobic metabolism, thermoregulation, and body temperature control have reduced amplitude in obese mice *Neotomodon alstoni*.

## Introduction

Circadian physiological responses anticipate daily environmental changes, such as light-dark or temperature cycles, so that predictive homeostasis in energetic balance favors survival. Circadian thermoregulation in mammals is achieved by changes in behavior and metabolism that compensate for heat fluctuations (1). In acute exposure to cold, mammal's heat production is regulated by changes in motor activity intensity and metabolic heat production by shivering as well as non-shivering thermogenesis, respectively (2). Mammal thermoregulation is under hypothalamic control. It is maintained through metabolic, energy, and endocrine adjustments (3) that may vary depending on the time of day, the season, sex, or age. Exposure to cold triggers a metabolic activation to preserve body temperature (BT) by an integrated response in diverse hypothalamic nuclei, such as the preoptic area and the dorsomedial hypothalamus (3, 4), complemented by the activity of the brown adipose tissue (BAT). This response is modulated along the day-night cycle by the arcuate and suprachiasmatic nuclei (5).

Mitochondrial uncoupling protein 1 (UCP1) is a functional protein that contributes to thermogenesis in Brown Adipose Tissue (BAT). UCP1 is produced by multiple pathways (6–8). Among the most important is the release of noradrenaline from the innervating nervous system in BAT; its synthesis is dependent on the enzyme tyrosine hydroxylase (TH), the limiting enzyme of catecholamines (9).

Obesity affects thermoregulation in different ways, i.e., by thermal insulation of enhanced subcutaneous fat, low heat production due to reduced motor activity (10, 11), endocrine dysregulation, i.e., thyroid (12) and leptin resistance (13). At the same time, obesity in mice negatively affects circadian rhythms; for example, the molecular circadian clock modulates metabolic mechanisms in active bioenergetic tissues, including BAT, whereas the regulation of BT changes according to the time of day (14, 15). Mechanisms affected by obesity in the circadian thermoregulatory responses still need research. A better understanding of the physiological and behavioral changes in different animal models adapted to face seasonal changes may enrich our knowledge of how different species of rodents handle thermoregulation.

The volcano mouse *Neotomodon alstoni* (Merriam, 1898), a nocturnal rodent of the Neotominae subfamily, genus *Peromyscus*, subgenus *Neotomodon* (16), it is restricted to the Transverse Neovolcanic Ridge of the central zone of Mexico, where inhabits pine forests, in grass lands. It can be found between 2,600 and 4,600 Meters above sea level (m.a.s.l.) (17). *N. alstoni* lives in burrows and feeds on grains and insects (18). Adult mice have a nasoanal length is 100–130 mm, ears are almost bare; hair of the dorsal region is dense and gray while the ventral fur is whitish. Adults usually weigh from 40 to 50 g (19). A cytogenetic analysis shows that its chromosome number is 2n = 48 with a fundamental number NF = 66, like *Peromyscus* (20). *Neotomodon alstoni* is listed as Least Concern species, according to the red list of the International Union for Conservation of Nature (IUCN) (21). It may adapt favorably to captive conditions (22, 23) and it can live up to 5 years in laboratory conditions (24). When captive or raised in vivarium conditions, part of the animals raised or captured spontaneously develop obesity when fed regular chow food, this characteristic seems to be congenital because potential conditions, other than *ad libitum* food access and isolation are equal for all mice, and intermediate phenotypes of pre-obese mice that suggest a heterozygous phenotype (25). Obese adult mice usually weigh more than 60 g and, compared with lean mice (BW~45 g), have alterations in the amplitude of the circadian rhythm of motor activity, as well as in the daily signaling of various hormones involved in metabolism such as insulin, leptin (26, 27) and ghrelin (28). The behavioral and endocrine changes previously observed suggest that obesity status in this species may also be associated with a deficit in the nictemeral behavioral and metabolic thermoregulation and its thermogenic response to a cold environment. We aimed to determine whether obese *Neotomodon alstoni* mice exhibit a deficit in metabolic rate and thermoregulation compared to lean mice over the 24 h profile in light/dark cycles (LD). In addition, we explored its possible relation with a change in the relative presence and gene expression of the mitochondrial Uncoupling Protein UCP1, and the relative presence of Tyrosine Hydroxylase (TH) in interscapular Brown Adipose Tissue (iBAT). Histological analysis of iBAT was also performed. Finally, to understand potential differences in thermoregulation defense in a cold challenge, we explored changes in body temperature when mice were exposed to a 2 h cold pulse, both at noon and midnight.

## Materials and methods

### Animals

Adult male mice *Neotomodon alstoni* were born and raised in the vivarium of Science Faculty at the National Autonomous University of Mexico (UNAM), as indicated elsewhere (29). Maintenance conditions included regulated photoperiod (Light: Dark cycles 12:12, 350–500 lx, lights on at 06:00 h), and controlled room temperature (23 ± 1°C). After weaning, mice grew individually in acrylic boxes with a metal grill lid and free access to regular rodent food (Purina, 5001) and tap water. Then, after 7–12 months, mice were separated into two groups: Lean (46 ± 3 g), and obese mice (65 ± 3 g) and used in experimental protocols indicated below. Each set of results indicates the number of animals used for every protocol. LD schedule is indicated in Zeitgeber time (ZT), considering ZT0 when the lights were on and ZT 12 when the lights were off.

Mice *Neotomodon alstoni* that show obesity can be distinguished from normal-weight mice when at ca. 7 months old (26, 27). Therefore, young adult mice in lean or obese groups were males between 7 and 12 months old. At the end of the protocols, mice were euthanized by decapitation, and interscapular adipose tissue was collected, kept in deep freezing, or prepared for histological analysis. All experimental protocols and procedures were performed following the Declaration of Helsinki and the Guide of the National Institutes of Health for the Care and Use of Laboratory Animals, NIH Publication No. 8023; guidelines and the General Health Law for Research Studies in Mexico (NOM-062-Z00-1999), based on ethical management for chronobiology studies (30) and the Committee on Academic Ethics and Scientific Responsibility of the Sciences Faculty, and the Neurobiology Institute UNAM.

### Indirect calorimetry

Indirect calorimetry was determined in intact lean (*n* = 10) and obese mice (*n* = 5) in a room with environmental conditions controlled (12:12 photoperiod, 06:00–18:00 h, photophase, 21 ± 1°C) at the Neurobiology Institute, UNAM. Respirometry parameters such as oxygen consumption (VO_2_), production of carbon dioxide (VCO_2_), respiratory quotient (RQ = VCO_2_/VO_2_), and energy expenditure (EE) were measured as indicated elsewhere (31) utilizing an OxyletPro system and the Metabolisms software v.3.0.01 (Panlab Harvard Apparatus, Barcelona, Spain). Mice were first acclimatized for 2 days in individual acrylic cages (Oxylet LE 1305 Physiocage, Panlab) with food and water. Then, respirometer parameters were obtained every 3 min for 3 days at a controlled flow rate of 700 ml/min (Oxylet LE 400—supplier air, Panlab). During all procedures, mice had access to food and water *ad libitum*, and both parameters were also monitored.

### Body temperature loggers

According to previous studies, intraperitoneal body temperature was recorded using the data logger iButton (Thermochron DS1921H-F50#; Dallas Semiconductor, Dallas, TX, USA) (32). It has been proven elsewhere that iButtons have been useful in studies using smaller mice CF1, BALB/c, and C57BL/6N (~35 g) (33) than in lean adut *Neortomodon* (~45 g). Loggers were programmed to record and store temperature data every 10 min, and recording was programmed to begin 10 days after implantation. Surgery was performed on anesthetized mice with intraperitoneal ketamine/xylazine (Anesket/Procin, Pisa, Mexico) 100/10 mg/kg weight. The abdomen's skin was shaved, and the area was cleaned with 70% ethanol and iodine solution. A longitudinal incision of ~2 cm was made along the midline, and the previously disinfected iButton covered with sterile wax (surgical specialties Co. Mx) was inserted into the abdominal cavity. The muscle and skin incision were separately sutured with nylon thread. After surgery, mice were kept on a warm bed until recovery from anesthesia; analgesia was provided by topic xylazine. Animals were allowed to recover from surgery for at least 10 days. Daily curation of the wound and cleaning cages were provided. Only mice that were full recovery from surgery with a locomotor activity pattern typical of intact animals were used for the temperature recordings. BT was monitored for at least 3 days (LD 12:12 at 23°C, LD 12:12 at 23°C with a 2 h cold pulse at noon and midnight). During the recordings the cages and substrate (clean wood chips) were replaced weekly. At the end of the experiment, mice were sacrificed by decapitation. The interscapular and gonadal adipose tissue was immediately frozen or preserved in paraformaldehyde, and their data loggers were removed to collect data using the iButton-Thermochron^®^ software.

### Control of environment and exposure to cold

Mice were kept in a controlled temperature environment using a Plant-Growth Chamber (Adaptis 1000, CONVIRON), which provided a stable and programmable temperature maintained at 23 ± 1.5°C, humidity 40%, with a photoperiod LD 12:12 using white LED light (300 lx). Then, cold exposure pulses were programmed as a drop in temperature from 23 to 10°C for 2 h; the pulses were programmed to take place at midnight (24:00–02:00 h) and noon (12:00–14:00 h) in LD 12:12 (06:00–18:00 h, photophase).

### Protein immunodetection by western blot

The relative presence of UCP1 protein was analyzed using Western Blot, as previously indicated (34). The iBAT samples from mice euthanized at noon were frozen in dry ice and kept at −80°C until analyzed. The adipose tissue was removed and homogenized in 4 volumes of RIPA buffer (Cell Signaling, Danvers, MA) supplemented with complete protease inhibitors (Roche Diagnostics, Indianapolis, IN) using a Potter-Elvehjem Teflonglass homogenizer (70 rpm for 15–20 s). The homogenate was centrifuged at 2900 rpm for 5 min at 4°C, and the infranatant (intermediate layer) was carefully recovered, aliquoted, and stored at −80°C until used. Total protein concentration was determined by the Bradford assay (Bio-Rad, Hercules, CA). Samples of 50 μg of protein mixed with 2X Laemmli buffer (Bio-Rad) and incubated at 80°C for 5 min were used. Proteins were separated on a 7.5% polyacrylamide gel, transferred to a nitrocellulose membrane (Bio-Rad), and blocked with Blotting-Grade Blocker (Bio-Rad) for 3 h. Then, the membranes were incubated separately overnight at 4°C with different rabbit antibodies (anti UCP1 EPR20381 ab 209483 Abcam dilution 1:1,000, and anti-Tyrosine Hydroxylase AB152 Sigma-Aldrich; dilution 1:5,000). The membranes were washed and incubated for 2 h with alkaline phosphatase (AP) conjugated antibody: donkey anti-rabbit IgG-AP (1:5,000 dilution; sc-2315; Santa Cruz Biotechnology, Dallas, TX, USA). According to the manufacturer's instructions, the bands were visualized using the AP conjugate substrate kit (Bio-Rad Laboratories, Hercules, CA, USA). Transfers were digitized, analyzed with Image J^®^ software (version 1.38, NIH, USA), and normalized to the α-tubulin signal (anti-α-tubulin ab 15246, Abcam, 1:1,000 or anti GAPDH 1:5,000 Abcam 9485) as a loading control.

### Hematoxylin-eosin staining

Interscapular and gonadal adipose tissue (epididymal) was fixed in paraformaldehyde 4% for 24 h, then transferred to a 10% formalin fixative solution for 1 week, and then to zinc-formalin (1% zinc sulfate) (35). Subsequently, the tissues were dehydrated for paraffin waxing, following the previous description indicated elsewhere (36), using a 12 h automated dehydration process in a Histokinette equipment (Leica TP 1020) at the Veterinarian Histopathology facilities of the Autonomous University of Querétaro, México. Tissue in 20 μm sections was counterstained with hematoxylin-eosin staining. Three slides from each sample of interscapular brown adipose tissue (iBAT) and gonadal WAT were taken from lean and obese mice (*n* = 3 from each group), and analyzed with Image J^®^ software (NIH, USA) to measure the size of gonadal adipocytes and the content in brown adipocytes (25 mm^2^), the average weight of interscapular adipose tissue was compared (lean *n* = 6, obese *n* = 7).

### *Ucp1* gene expression by RT-qPCR

Total RNA was isolated from *Neotomodon alstoni* from iBAT, quickly dissected and frozen in dry ice and kept at −80°C until further processing (37). We use the TRIzol™ Reagent (Invitrogen Life Technologies, Carlsbad, CA) following the manufacturer's instructions, with minor modifications (38, 39). Genomic DNA contamination was removed by treating 1 μg of total RNA per sample with 10 units of DNase I (#04716728001; Roche, Basel Switzerland), and cDNA was synthesized using the High-Capacity cDNA Reverse Transcription Kit with RNase Inhibitor (#4374966; Applied Biosystems™ Inc., Foster City, CA) following the manufacturer's instructions. We included negative controls without a template or reverse transcriptase, cDNA was diluted 1:5 before use.

Currently, the genome sequence for non-traditional animal model *N. alstoni* mice is not available. However, from a previous study (16) upon comparative taxonomy of muroid species *Peromyscus maniculatus* and *N. alstoni*; primers were designed using GenBank® genetic sequence database for *Peromyscus maniculatus bairdii*, using NCBI Primer-BLAST, for ucp1 gene [Uncoupling protein 1 (mitochondrial; proton carrier) mRNA; Accession Number: XM_006995737.2] and for tbp gene (TATA-box binding protein mRNA; Accession Number: XM_042261953.1), for normalization of mRNA expression (40). All primers were designed to span exon-exon boundaries and were analyzed *in silico* with NetPrimer (PREMIER Biosoft International, Palo Alto, CA) software). Oligonucleotide primer sequences are given in Table 1.

**Table 1:** Oligonucleotide primer sequences for RT-qPCR.

**Primer name**	**Synthesis direction**	**Sequence (5′-3′)**
naUcp1	Forward	ACACTCTGGAAAGGGACAAGC
	Reverse	CACACTTGGGTACTGTCCCG
naTbp	Forward	GGCATCACTGTTTTATGGTGTGT
	Reverse	GGAGTCATGGCACCCTGTG

All real-time PCR reactions were carried out in the StepOne™ Real Time System (ThermoFisher Scientific, Waltham, MA), using KAPA SYBR^®^ Fast qPCR Kit (KAPA BIOSYSTEMS, Woburn, MA, USA), according to Martínez-Moreno et al. (41). The reactions were performed in duplicate under the following conditions: initial denaturation at 95°C for 3 min followed by 45 cycles (*Ucp1*) or 40 cycles (tbp) of 95°C for 3 s, 61°C (ucp1) or 62°C (*tbp*) for 20 s and 72°C for 10 s. A dissociation curve was run to ensure that there was only one specific amplification product for NTC or negative controls; there was no detection signal. cDNAs standard curves were prepared using serial dilutions to obtain the primer efficiencies, which were 90% for ucp1 and 90.5% for tbp. We used a relative standard curve quantification method (42, 43) to normalize *Ucp1* mRNA expression levels between experimental lean and obese mice groups, to the mRNA level of the reference gene *tbp*, by generating standard curves for each gene using pooled cDNA.

### Data analysis

The results are expressed as mean ± SEM of each parameter. For statistical analysis and plotting, we used the GraphPad Prism 7.0 (GraphPad Software Inc., San Diego, CA, USA). Intergroup comparisons were tested with a two-way ANOVA for independent measures with a factor for each group, a factor for time, and a Fisher *post-hoc* test. To assess the mean total area under the curve (AUC) in the 24 h daily profile, a comparison of the number and size of adipocytes and relative expression of proteins from iBAT between lean and obese mice was performed with a Mann-Whitney U-Test. Normalized U*cp1* mRNA expression in BAT of relative quantification method from RT-qPCR data was averaged and compared between groups by non-parametric Mann Whitney *U*-Test. For all analyses, statistically significant differences were considered at *P* < 0.05.

## Results

### Metabolic rate

**Figure 1:**
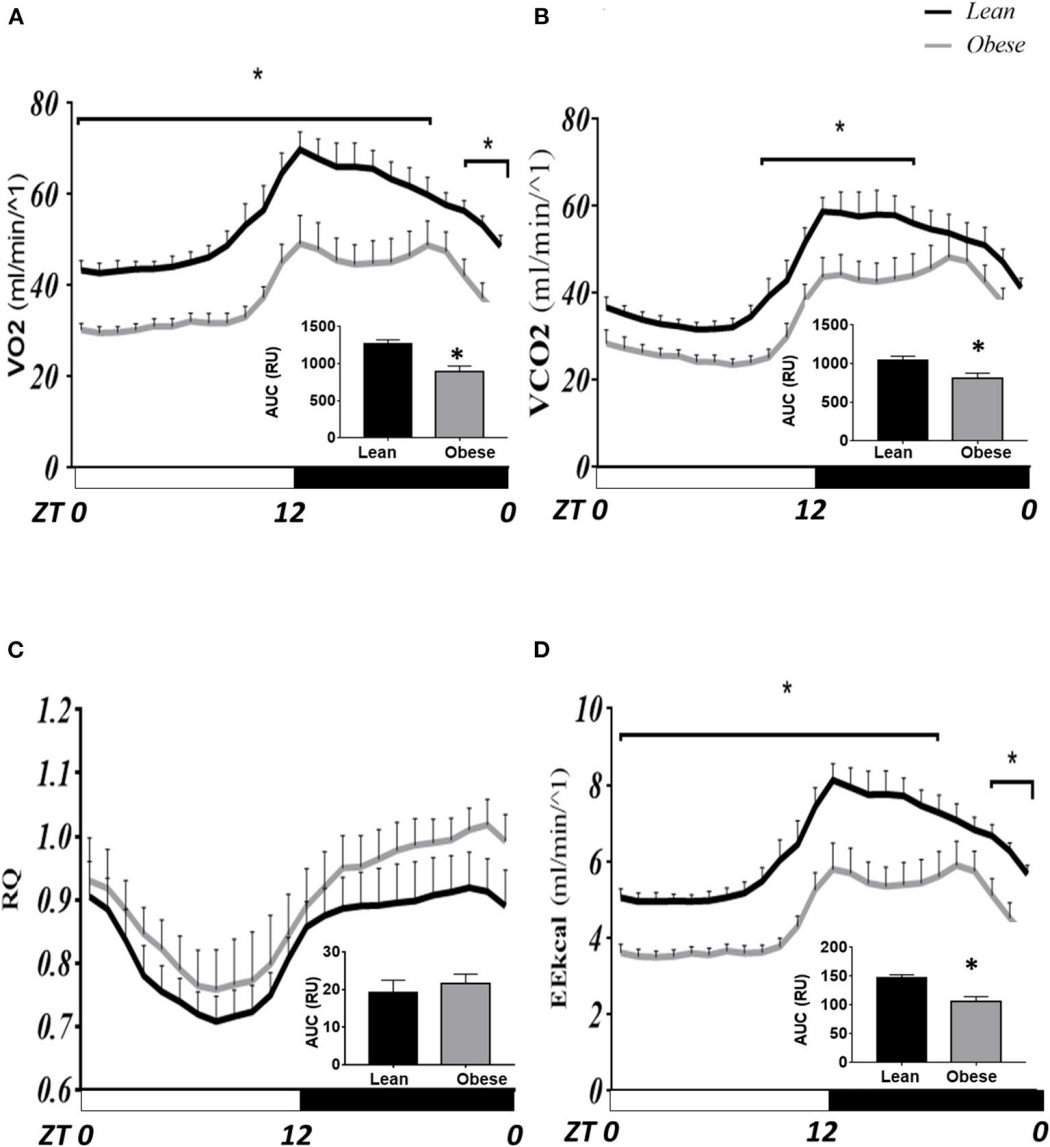
Comparative daily profile of calorimetry parameters in mice *N. alstoni*. Data are presented as mean ± SEM, *n* = 10 lean, *n* = 5 obese. Lean (black line), Obese (gray line). Bars depict the LD cycle. *Significant differences intergroup (two-way ANOVA, *P* < 0.05). **(A)** VO_2_ intake; **(B)** VCO_2_ production; **(C)** Respiratory ratio; **(D)** Energy Expenditure (kCal).

Figure 1 shows the hour-by-hour mean ± SEM along the LD cycle on the parameters evaluated in intact mice inside metabolic cages on LD 12:12. The VO_2_ and VCO_2_ profiles (ml/min^−1^, Figures 1A,B, respectively) show a daily rhythm, with a rise before lights off, fades throughout the night, and falls before the onset of lights. Obese mice display a lower volume of oxygen intake and CO_2_ production in most of the schedules tested. The bimodal pattern shown by the obese mice during the night is more consistent with previously observed locomotor activity patterns (34). Significant differences found between groups simultaneously are indicated with a star (*P* < 0.05). The hourly average VO_2_ intake was not different only at ZT 20 and 21. In contrast, VCO_2_ production was different over several hours, between ZT 10–18, corresponding to the end of the photophase and the first half of the scotophase. The AUC was lower (31%) in obese VO_2_ intake (893.4 RU) than in lean mice (1,295 RU, *P* < 0.05); while in CO_2_ production, AUC in obese was lower (26%) (797.6 RU) than in lean mice (1,077 RU, *P* < 0.05), indicating low aerobic metabolic demand in obese mice (inside graphs).

In Figure 1C, the Respiration Coefficient (RQ) tends to be higher in obese than in lean mice; although, no significant differences were detected in these parameters between groups nor in AUC. However, energy expenditure (EE kcal/min-1) was different at most times of the day except for ZT 21 and 20, similar to VO_2_ intake (Figure 1D). The AUC for EE showed a similar pattern to that for VO_2_ intake, where obese mice had lower energy expenditure (21%) (3,177 RU) than lean mice (4,041 RU; *P* < 0.05).

**Figure 2:**
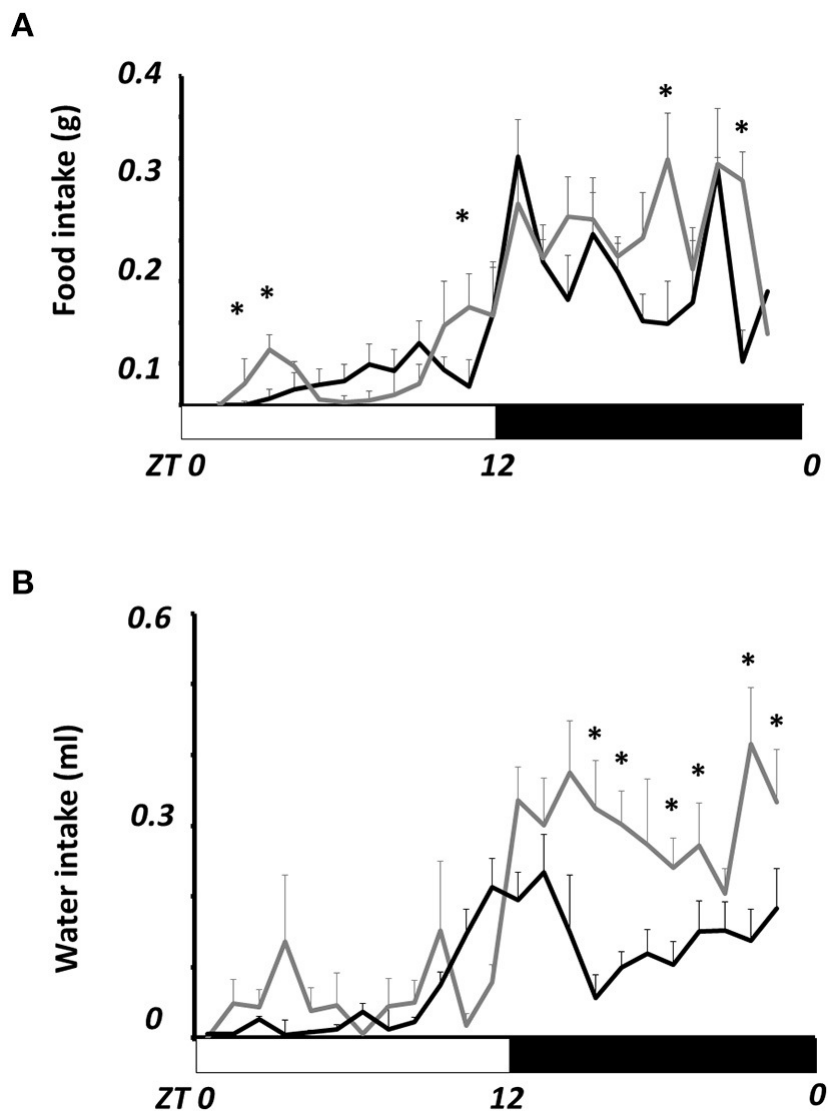
Comparative daily profile of daily food **(A)** and water **(B)** intake in lean and obese N. alstoni. Data are presented as mean ± SEM, *n* = 10 lean, *n* = 5 obese. Lean (black line), Obese (gray lines). Bars depict the LD cycle. *Significant differences intergroup (two-way ANOVA, *P* < 0.05).

Figure 2A shows the daily profile of food intake. The average intake in obese mice (5.8 ± 0.7 g/day) was not statistically different from lean mice (5.0 ± 0.4 g/day). However, obese mice eat more than lean mice during the day at ZT 1, 2, and 9; and at ZT 19 and 22 during the night (*P* < 0.05). Water intake (Figure 2B) was not different during the day, but it was mainly during the night, from ZT 15- to ZT 23, where obese mice drank nearly twice more water than lean mice (*P* < 0.05).

### Body temperature and response to cold exposure

**Figure 3:**
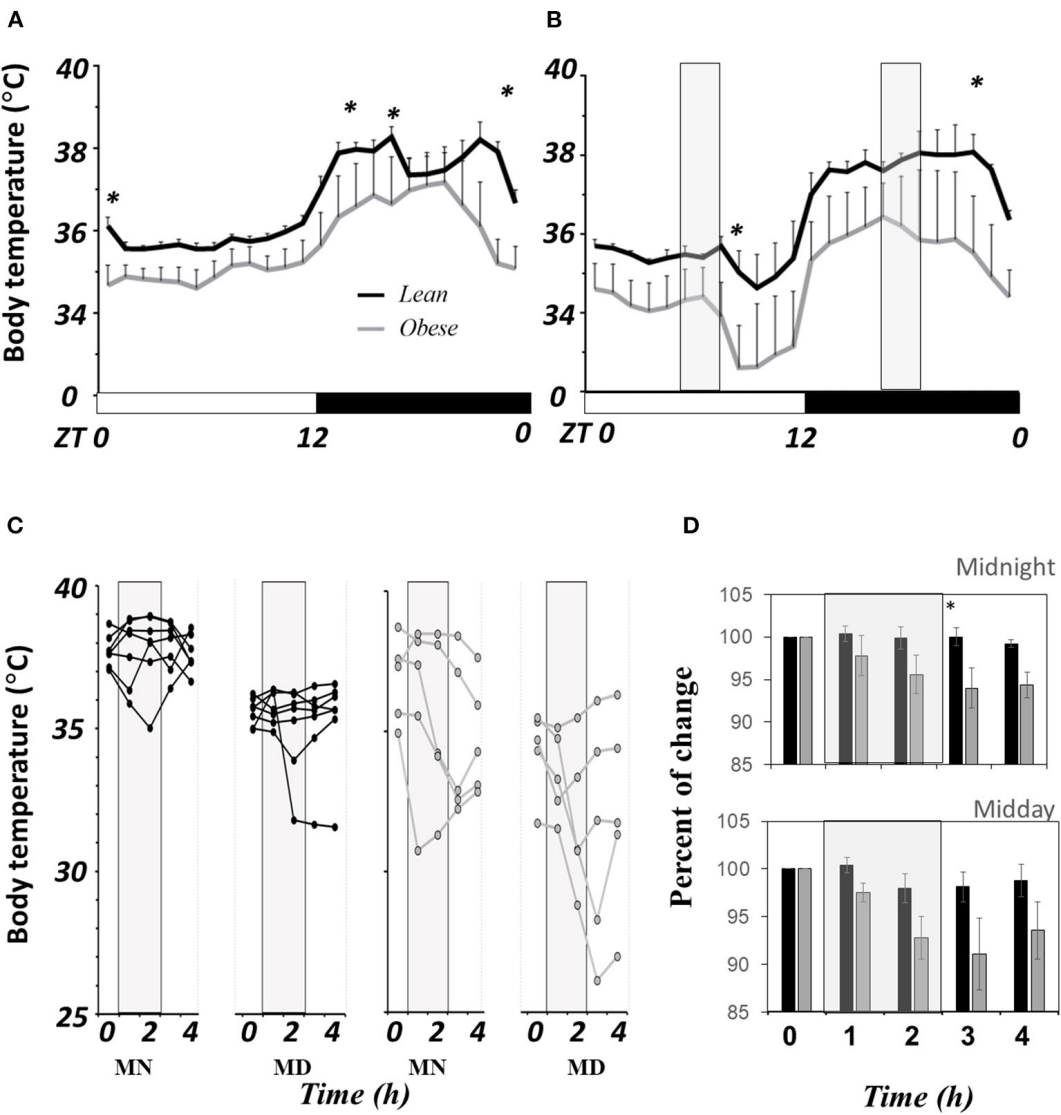
Comparative of core body temperature in *N. alstoni*. Lean (black lines and bars, *n* = 7), Obese (gray lines or bars, *n* = 5). **(A)** Hour by hour average ± SEM of 3 days in LD; **(B)** Daily profile temperature and exposure to a drop in environment temperature (12°C, light gray bars), **(C)** first day individual changes in BT before during and after the cold exposure; **(D)** Percent change of body temperature before, during and after cold pulses vs. Time 0. *Significant differences intergroup (*P* < 0.05).

Figure 3A shows the 3-day average (±SEM) of body temperature (BT) in LD from lean (black lines, *n* = 7) and obese mice (gray lines, *n* = 5) in a 24h LD profile. Significant differences between groups are noted in brackets (*P* < 0.05). Obese mice show lower BT than lean mice at onset and offset of the scotophase. Average BT in obese mice during the day was lower (34.9 ± 0.06°C) than in lean mice (35.7 ± 0.06°C), a reduction of 2.4%; while during the night, obese mice presented 36.4 ± 0.20°C, and in lean mice 37.6 ± 0.1°C, a reduction of 3.5%, nearly 1°C below the average in day and night (*P* ≤ 0.05). The AUC was smaller in obese mice (820.9 ± 13.7 RU) than in lean mice (844.9 ± 2.4 RU), with no significant differences.

When exposed to cold in a 2-h drop from 25 to 10°C in ambient temperature, the thermoregulatory response was evidently different between lean and obese mice. Figure 3B shows the 3-day average of BT's daily profile (mean ± SEM), and gray bars indicate the time when the ambient temperature falls (cold pulses). Differences in BT were found right after the pulse at either noon or midnight. The change in BT during the first day of the protocol is shown in Figure 3C. Data from each mouse are plotted with a line in black (lean *n* = 7) and gray (obese mice *n* = 5) before the pulse (time 0), then during the first and the second hour during the cold pulse (hours 1 and 2), and after the pulse (hours 3 and 4), respectively. The results showed a variable response mainly among obese mice; some showed a clear drop in BT during the pulse that remains low after the pulse, while few obese mice could defend BT from cold. At midnight, lean mice (black dots) compensated BT from the first hour of cold exposure, and at noon, only one out of six mice showed a drop in BT. The average (±SEM) of the percent change regarding the time 0 is shown in Figure 3D. Even though there is a fall during the pulse regarding the time 0, there are no significant differences between lean (black bars) and obese mice (gray bars) until after the cold pulse finished. Obese mice reduced their BT by nearly 10%, and the change seems to be persistent 2 h after cold exposure.

### Relative expression of the protein UCP1, TH, and histology of interscapular adipose tissue

**Figure 4:**
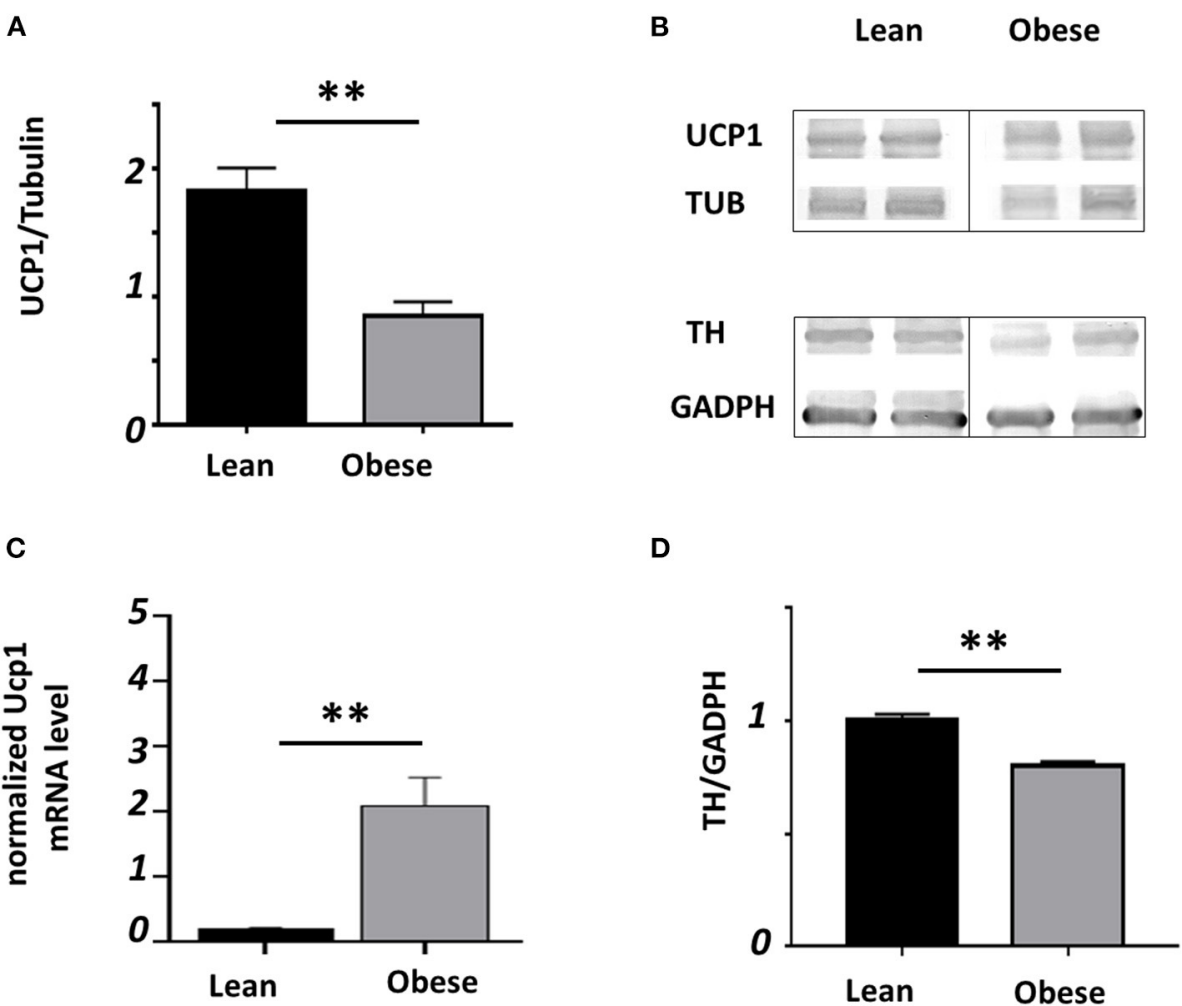
Relative presence of UCP1 and TH in iBAT. Black bars represent lean and gray bars obese mice. **(A)** Relative presence to tubulin lean (*n* = 14) and obese (*n* = 8). **(B)** Representative blots. **(C)** Relative mRNA for Ucp1 (*n* = 6 lean, *n* = 4 obese) analyzed by RT-qPCR and normalized to the reference gene tbp. **(D)** Relative abudance of TH (*n* = 5 each group); (**P < 0.01).

Figure 4A shows the mean ± SEM of the relative presence of UCP1 detected by WB with an α-tubulin signal as the protein of reference from samples of iBAT (*n* = 14 lean, *n* = 8 obese mice) collected at noon. Representative blots are shown in the right panel (Figure 4B). Obese mice showed nearly 50% less relative presence of UCP1 than lean mice, which may be related to a thermoregulatory deficiency seen before, shown in Figure 3C. Unexpectedly, the relative expression of normalized *Ucp1* shows a nearly 11-fold increase in obese (*n* = 4) than in lean (*n* = 6) mice (*P* < 0.01; Figure 4C). On the other hand, the protein thyroxine hydroxylase relative to GADPH in iBAT evaluated by Western Blot (Figure 4D) show a reduction of nearly 20% in obese mice (*n* = 5, each group).

**Figure 5:**
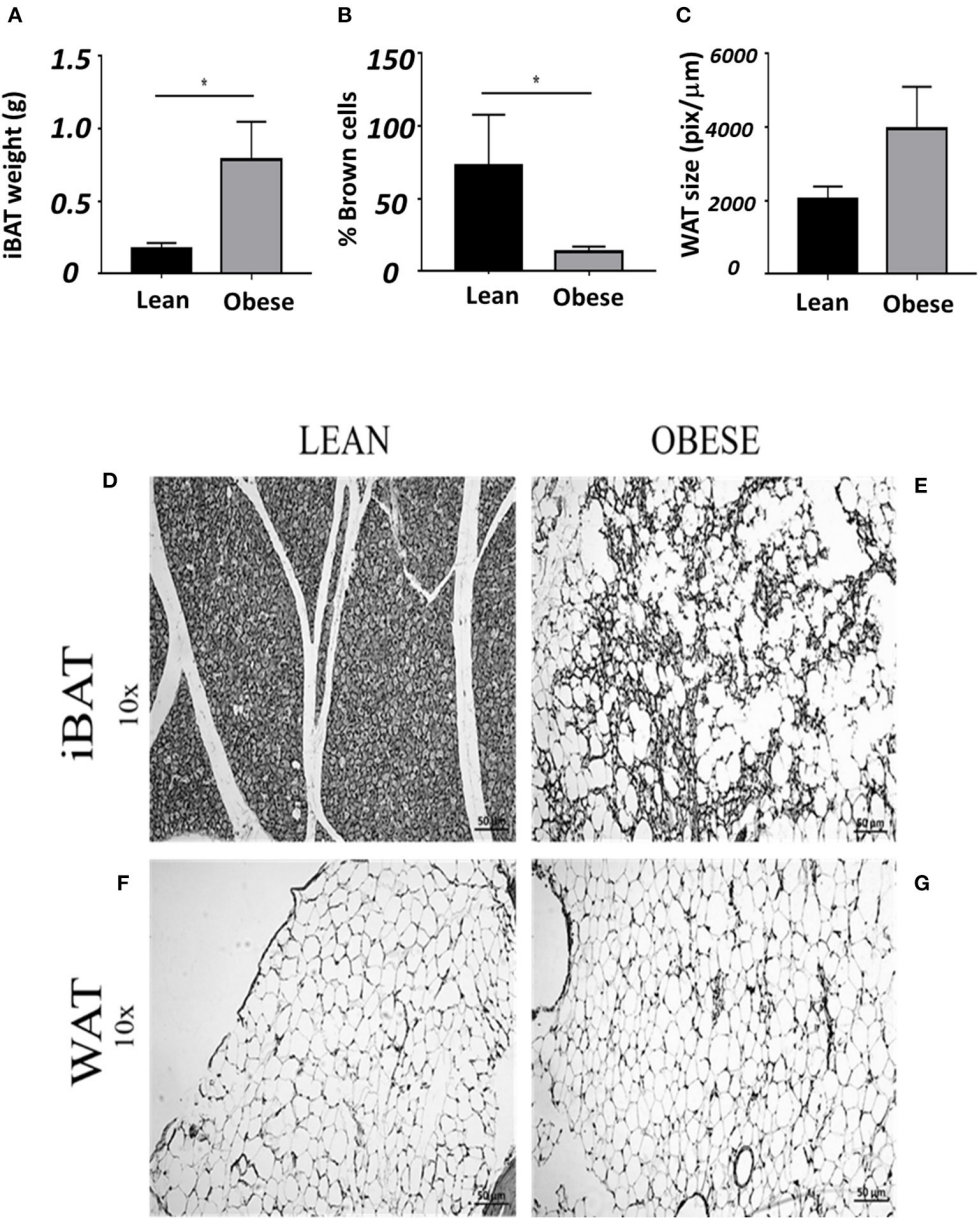
Average ± SEM of: **(A)** weigh of iBAT, **(B)** % of brown adipocytes **(C)** size of white gonadal adipocytes. **(D)** Slide of iBAT from a lean, **(E)** obese iBAT. **(F)** Gonadal white adipose tissue from lean and **(G)** obese mice. *Significant differences intergroup lean and obese male mice (*P* < 0.05).

Figure 5 shows the quantitative histological analysis of slides contrasted with hematoxylin and eosin of staining in samples from iBAT and compared with gonadal white adipose tissue (WAT). Obese mice (gray bars) had nearly 4 times more adipose tissue than lean mice (black bars) at the interscapular zone (Figure 5A). Lean mice had a brown compact mass of adipocytes (Figure 5D). In contrast, obese mice showed adipose tissue with a lighter color (white-yellowish) than that of lean mice, consistent with the low proportion of brown adipocytes observed on slides (Figure 5E). The iBAT in obese does not show abundant blood vessels as it is in lean mice. The histological composition of iBAT in lean mice is mainly composed of brown adipocytes (small cells in dark gray), contrary to obese mice, in which brown adipocytes are scarce (nearly 75% less than in lean mice, Figure 5B). White adipocytes tend to be larger in gonadal adipose tissue of obese than lean mice (Figures 5C,F,G).

## Discussion

In vertebrates, thermoregulatory needs are closely related to energy balance, and endothermic animals always prefer normo-thermal conditions to reduce energy costs. Obesity in mammals has an influence on thermoregulation in different ways, increasing the thermal insulation conferred by subcutaneous fat (10), reducing motor activity (11), as well as modifying endocrine thyroidal metabolism (12) and leptin signaling (13). Thermoregulation in obese rats is lower than in lean rats (44), and studies about the metabolic rate and obesity in rodents rely on models where the genetic structure or diet is modified to highlight the overweight condition (45, 46).

The present work describes the difference in daily thermoregulation and energy metabolism between lean and obese *N. alstoni* mice. This species is interesting because spontaneous obesity occurs in some mice raised in captivity, isolated in cages, and with *ad libitum* access to bioterium food. The biological causes for developing this condition are still uncertain. Along with previous studies [reviewed in (25)], this work helps to understand the circadian affectations in behavior, metabolism, and thermoregulation in obese mice. It is also relevant in line with circadian dysregulations related to the obesity condition, such as reduced locomotor activity, alterations in glucose regulation, oxidative stress, leptin, and ghrelin signaling, as well as hepatic lipid dysregulations that may be linked to metabolic disorders.

In the present work, we show that in *N. alstoni*, metabolism and thermoregulation are down-regulated in obese mice. Indirect calorimetry assays showed that the main differences between lean and obese mice occur in the ratio VO_2_/VCO_2_ throughout the day. However, the RQ showed no differences, and we cannot conclude that different energetic substrates may be differentially used in obese mice. Nonetheless, a tendency to be higher in obese mice suggests that more carbohydrates are used instead of lipids during the night. The RQ involves Vol CO_2_ released/Vol O_2_ absorbed; carbohydrates are oxidized through aerobic respiration resulting in an equal ratio of VCO_2_/VO_2_ (RQ = 1). Subsequently, the RQ for fat and protein metabolism is 0.7 and 0.8, respectively. If a mixture of the substrates is consumed, the RQ ratio collectively is ~0.8 (47). Results in Figure 1C suggest that lipids are more likely to be used during the rest phase of the day in lean mice, and obese mice may have to use a mixture of substrates. This interpretation could be related to the excess of adipose tissue detected in obese mice.

Energy expenditure (EE) in kCal/min^−1^ (Figure 1D) was clearly reduced in obese mice. In previous studies, we observed that obese *N. alstoni* mice show half the amount of locomotor activity during the night and sometimes more activity during noon than lean mice (27, 37), results that are consistent with a reduced EE observed during the night in the present work. Nevertheless, during the rest phase, EE in obese mice is still reduced compared to lean mice, indicating that metabolic use of energy during rest is also different, as observed in body temperature results (Figure 3A) as well as it may be related with the enhanced food intake observed during the day in obese mice (Figure 2A). Also, polydipsia observed in obese mice (Figure 2B) could be related to a diabetogenic tendency detected previous reports (26), since it is recognized that diabetic organisms present polydispsia (48). The diabetic db/db mice present a spontaneous mutation of the leptin receptor (49) also show polydipsia as well as increased food intake. Eating and drinking is an activity mediated by lateral hypothalamic area (LHA) and a subpopulation of LHA neurons expressing Neurotensin also present the leptin receptor (50). LHA neurons may promote water intake in mice (51), in *Neotomodon* reduced hypothalamic leptin receptors and leptin transduction previously observed (27) may affect the leptin regulation of water intake.

Reduced BT in obese mice was on average, almost 1°C colder than that observed in lean mice, while during the active phase, there were times when the difference reached even 3°C below, such as the end of the night. These data suggest that obese mice might present reduced oxidation of fat deposits and thus lower heat production, implying a different homeostatic set point. A similar response has been previously observed in ob/ob mice. However, it has been discussed that such differences in thermoregulation may not include a shift in the homeostatic set point (52).

Metabolic phenotypes of obese mice may be useful to better understand the relationship between genetic disorders or physiological processes and energy metabolism *in vivo*. Understanding thermoregulatory alterations across different thermic challenges may reveal specific deficiencies in responses to cold and heat challenges (53). In obese *N. alstoni* mice, the exposure to a cold pulse did not change body temperature in lean mice, but a diversity of responses was observed in obese mice, especially during the resting phase. A decrease in body temperature in obese mice could be related to a possible facilitation of torpor in obese animals; this hypothesis may be tested by analyzing the circulating pyruvate in obese mice after a cold pulse that seems to facilitate the torpor (54). Daily torpor facilitates survival when animals are faced with limited energy resources, usually in adverse environments. In other *Peromyscus* species, torpor occurs when starvation is sufficient to significantly reduce body weight, and this is most notable in animals that fatten under laboratory conditions (55), as well as ob/ob obese mice (56). In obese *Neotomodon alstoni*, restricted access to feed for 4 h during the day produces a significant reduction in feed intake and body weight, but does not significantly reduce motor activity (57) or induce torpor. Obese *Neotomodon* seems to be more sensitive to cold rather than lack of food. The possible resistance to leptin in obese *Neotomodon* (27) could be involved in the torpor response to cold. In ob/ob mice, leptin has been shown to reduce the torpor response to a period of fasting (58).

The reduced thermoregulatory response appears to be linked to a deficiency of the UCP1 protein in iBAT (Figure 4A), which could be related to the different proportions of brown and white adipocytes in lean and obese mice. It has been demonstrated that transcription of the UCP1 gene is up-regulated by adrenergic stimulus (59), in our experiments, we found low presence of TH in obese mice. The UCP1 is a member of the mitochondrial carrier protein superfamily, all of which have similar molecular weights and structural similarities (60). We used a monoclonal antibody specific for rat and mouse UCP1 protein; however, we do not know if it recognizes *Neotomodon*'s protein; therefore, we cannot exclude the possibility that the antibody recognizes any of the UCP1 members of the mitochondrial superfamily. To understand whether *Ucp1* gene expression was proportional to the relative protein, we performed the mRNA analysis using a sequence for a phylogenetically related species *Peromyscus maniculatus*. BLAST analysis of the *Ucp1* sequence shows only one gene for *Peromyscus*, as for *Mus musculus*. As long as there is a lack of information in *Neotomodon*'s genome, approximation of these mechanisms at the genetic level depends on their homology with the closest species. The qPCR results indicate an elevation in the relative presence of *Ucp1* mRNA in obese mice, which does not correspond to the discrete relative presence of the protein. Despite the few samples analyzed (*n* = 5 for each group), the observed differences were striking. This result indicates that the canonical pathway of adrenergic induction of UCP1 might not be involved in such expression and it will be necessary to explore various transcription factors such as Pgc1α, Prdm16, and Pparα (7) to better understand the regulation of *Ucp1* expression in obese mice. UCP1 is also regulated either at the transcription of gene and protein activity in mitochondria as well as its possible regulation by cAMP, retinoids, thyroid hormone, and other factors (6). In this scenario, our data could be explained by events that need further studies: (a) a strong inhibitory regulation of the traslational activity (at ribosomal level, nuclear transport, etc.), (b) a fragility or rapid exchange of the synthetized protein (covalent modifications, intracellular transport, etc.). In order to understand the difference observed in mRNA and protein, future research is required.

Brown adipose fat is innervated by the sympathetic nervous system. Adrenergic-stimulated breakdown of triglycerides in the lipid vesicles of the brown adipocyte provides rapid activation of thermogenesis, followed by the efficient distribution of heat released from brown fat for delivery to vital organs (61). A first approximation considering TH relative presence in iBAT indicates a slight drop in TH presence in iBAT, which is in line with the decrease in UCP1 protein. It is possible that in obese *N. alstoni*, reduced sympathetic tone may be a consequence of leptin resistance in the hypothalamus. Obese *N. alstoni* show high circulating leptin, while there is low expression of leptin receptors in the hypothalamus and their correspondent activity (27). Leptin can modulate thermogenesis and regulate energy expenditure. The action of leptin on LepRb neurons in DMH/DHA and mPOA mediates sympathetic tone (11), which in turn regulates the metabolic activity of brown adipose tissue, mainly due to UCP1 activity.

In addition, this process allows heat production and its dissipation (derived from non-shivering thermogenesis) with the release and use of fatty acids from the brown adipose tissue (62, 63). Sensitivity to the thermoregulatory effects of leptin appears to be integrated in part at the level of DMH (64).

The physiological basis of the substantial differences in metabolic efficiency between lean and the obese mice requires further study and may play a key role in understanding the pathophysiology of obesity (65). The plasticity of the adipose tissue is well known; recent research provides insight that BAT undergoes “bleaching,” a brown-to-white conversion induced by lipase and leptin receptor deficiency, as well as impaired β-adrenergic signaling, leading to brown adipocyte expansion, death, and obesity-related inflammation (66). Furthermore, BAT malfunction and tissue inflammation, including macrophage activity, appear to decrease thermogenic expression by low sympathetic innervation and norepinephrine activity (67). In the present work, we observed that obese iBAT exhibit large numbers of adipocytes resembling white adipose cells, suggesting similar bleaching. Also, low relative TH expression may be related to low sympathetic innervation and norepinephrine activity.

Obese *Neotomodon* resembles the ob/ob mouse deficient in leptin production (68). The ob/ob mouse is about three times the body mass of the lean mouse while its daily food intake is similar. At ambient temperature BT in obese is ~1–2°C lower than in the lean mice and presents a reduced capacity for thermogenesis due to exposure to cold without prior acclimatization (69, 70). Hypothermia in the ob/ob mouse is the result of a deficiency in heat production in brown adipose tissue (70, 71) consistent with a deficit in noradrenergic stimulation (72). On the other hand, mice with a leptin receptor deficiency (db/db) show also reduced energy expenditure in thermoregulation and thermogenesis such as in ob/ob mice (73). The deficit in noradrenergic stimulation of subcutaneous white and brown adipose tissue appears to be a consequence of a lack of leptin signaling in neurons expressing brain-derived neurotropic factor in the paraventricular nucleus of the hypothalamus (BDNFPVH), a descending neural pathway that it is crucial for energy homeostasis (74). The obese *Neotomodon alstoni* present hyperleptinemia, more clearly in females (26) and low leptin signaling in the hypothalamus (27). Given that in *Neotomodon* the obesity condition occurs spontaneously only in a subset of the captive mice, may be cause of some genetic deficit related to leptin signaling or leptin resistance that in this species still needs to be identified.

Although the causes may be multiple, it is essential to further investigate the relationship between thermoregulation deficit and obesity. For example, a deficit in TRPM8 cold receptors has been associated with low BT in obese mice (75). Furthermore, when given a cold pulse, lean Neotomodon showed a greater burst of locomotor activity than obese mice (data not shown), which involves the reduction of producing extra heat through exercise.

Obesity is the consequence of larger energy intake than energy expenditure. We noted in *N. alstoni* that mice eat more food. It is possible that the obesity in these mice may be a combination of a reduction in energy expenditure with a higher energy intake, considering that obese mice show reduced BT, locomotor activity, and low thermogenesis to compensate for a cold pulse than in lean mice, and less lipid oxidation during fasting; however, at this stage, we cannot distinguish which is cause and which is consequence.

Also, calorimetric assessment suggests that metabolic pathways regulating energy substrates and circadian organization of metabolism are impaired, and lipid catabolism is impaired in obese *N. alstoni* mice (34). The above is consistent with increased free fatty acids (FFA) and out of phase or reduced relative expression of hepatic PPARs, suggesting a potential enhanced lipolysis, but also possible deficient FFA uptake and/or reduced fatty acid oxidation in peripheral organs. A decreased metabolism and reduced heat production appear to be part of a circadian disruption in obese mice (76, 77). Obese animals are regulating their body temperature at a lower level than lean animals and are unable to maintain a stable body temperature in the face of a cold challenge. However, they are still able to maintain a daily rhythm; therefore, it does not appear to be a severe circadian dysfunction, but a rather a putative different set point in regulation. The causes of such change suggest hypothalamic differences that may target nuclei controlling thermoregulation, i.e., a function of heat-sensitive neurons in the preoptic anterior and dorsomedial hypothalamus that directly control the dissipation of heat (78).

## Data availability statement

The raw data supporting the conclusions of this article will be made available by the authors, without undue reservation.

## Ethics statement

The animal study was reviewed and approved by Bioethics Committee, Neurobiology Institute, and the Committee on Academic Ethics and Scientific Responsibility of the Sciences Faculty UNAM.

## Author contributions

Conceptualization: MM-A, MD-M, and AH-G. Methodology: MM-A, AH-G, MP-M, EA-L, DG-M, and AC-C. Formal analysis: AH-G, MM-A, and DG-M. Resources: MM-A and MD-M. Review and editing: All authors. All authors contributed to the article and approved the submitted version.

## Funding

This work was supported by the Dirección General de Asuntos Del Personal Académico (DGAPA, PAPIIT IN200620, and IN202121) to MM-A and MD-M.

## Conflict of interest

The authors declare that the research was conducted in the absence of any commercial or financial relationships that could be construed as a potential conflict of interest.

## Publisher's note

All claims expressed in this article are solely those of the authors and do not necessarily represent those of their affiliated organizations, or those of the publisher, the editors and the reviewers. Any product that may be evaluated in this article, or claim that may be made by its manufacturer, is not guaranteed or endorsed by the publisher.
